# Osteoconductivity of Bovine Xenograft Granules of Different Sizes in Sinus Lift: A Histomorphometric Study in Rabbits

**DOI:** 10.3390/dj9060061

**Published:** 2021-05-31

**Authors:** Eduardo Pires Godoy, Karol Alí Apaza Alccayhuaman, Daniele Botticelli, Andrea Amaroli, Vitor Ferreira Balan, Erick Ricardo Silva, Samuel Porfirio Xavier

**Affiliations:** 1 Department of Oral Biology, Faculty of Dentistry of Ribeirão Preto, University of São Paulo, São Paulo 14040-904, Brazil; eduardo.godoy@usp.br; 2 Department of Oral Biology, Medical University of Vienna, 1090 Vienna, Austria; caroline7_k@hotmail.com; 3 ARDEC Academy, 47923 Rimini, Italy; daniele.botticelli@gmail.com; 4 Department of Orthopaedic Dentistry, Faculty of Dentistry, First Moscow State Medical University (Sechenov University), 119991 Moscow, Russia; 5 Department of Oral and Maxillofacial Surgery and Periodontology, Faculty of Dentistry of Ribeirão Preto, University of São Paulo, São Paulo 14040-904, Brazil; vitor.balan@usp.br (V.F.B.); erickricardo.rp@gmail.com (E.R.S.); spx@forp.usp.br (S.P.X.)

**Keywords:** animal study, sinus floor elevation, bone healing, osteoconductivity, histology, morphometry, collagen membrane, xenograft

## Abstract

Background: Due to the lack of data on bone-to-graft contact (BGC) over time in the various regions within the subantral space of the augmented sinus floor, the present study aimed to evaluate the osteoconductivity of deproteinized bovine bone mineral (DBBM) with granules of different sizes applied in maxillary sinus floor elevation. Methods: A maxillary sinus augmentation was performed bilaterally in 18 rabbits using DBBM with particle dimensions of either 0.125–1.0 mm or 1–2 mm. The antrostomy was covered using a collagen barrier. The animals were euthanized in groups of six after 2, 4, and 8 weeks of healing. MicroCT and histological analyses were performed. Results: After 2 weeks of healing, BGC was 10.9% and 11.9% for the small and large granule sites, respectively. After 8 weeks of healing, the BGC increased to 65% and 62% at the small and large granule sites, respectively. The highest values were located close to the bony walls and the bony window. New bone content developed between 2 and 8 weeks from 7.0% to 27.6% and from 6.1% to 27.6% at the small and large granule sites, respectively. Conclusions: Similar outcomes in osteoconductivity and bone formation were found at both small and large DBBM granule sites.

## 1. Introduction

When the bone volume in the posterior regions of the maxilla does not allow the installation of implants of adequate length, a sinus floor augmentation procedure is often applied to increase the height of available bone. Due to the tendency of the sinuses to re-pneumatize over time after the elevation of the Schneiderian membrane [1,2,3,4,5], various biomaterials have been applied, aiming to counteract that re-pneumatization [6].

Xenografts of various particle sizes are widely used as filler materials [7,8,9,10]. Depending on their structure, the particles of xenografts will either be resorbed over time at different rates or embedded into newly formed tissues [11,12,13,14]. Deproteinized bovine bone mineral (DBBM) has been applied in several clinical [7,8,15,16] and experimental studies [11,17,18,19], showing high volumetric stability compared with other biomaterials.

The influence of the size of the particles on the clinical outcomes has also been evaluated.

In a randomized clinical study [20], a ridge preservation technique was applied after molar extraction. Human demineralized bone matrix putty was used as a filler. The putty either contained particles of small size (0.125–0.710 mm) or a mixture of larger dimension (2–4 mm) and small particles. No clinical or histological statistically significant differences were found between the two augmentation sites.

In a randomized controlled split-mouth clinical study [16,21], 10 partially edentulous patients were recruited for a bilateral sinus floor augmentation. Granules of different sizes (0.125–1.0 mm or 1–2 mm) of DBBM were used to fill the elevated space in the sinus. After 8 months of healing, 25 implants were installed, and biopsies were retrieved for histological analysis. No statistically significant differences were found either in terms of implant stability, measured after a further 6 months of healing, or regarding residual biomaterial and newly formed bone proportions.

These results are in agreement with the outcomes of other clinical [22] and animal [17] studies on sinus floor augmentation. However, a further clinical study [8] reported larger amounts of new bone formation, compared with small particle size sites.

The integration of DBBM particles (bone-to-graft contact; BGC) has also been evaluated in augmented sinuses [11,17]. In a study in minipigs [17], only the total BGC was evaluated, without providing data divided for each region. In a study in rabbits [11], BGC in various regions of the augmented sites was evaluated after different periods of healing. However, only sites with small granules of DBBM were evaluated.

Due to the uncertainty in the selection of the dimensions of the xenograft to be used and the lack of information about the osteoconductivity of large granules studied at different sites of the augmented sinus, the study of the influence of dimensions of xenograft particles on healing outcomes appears to be justified.

Hence, the present experimental study aimed to evaluate the osteoconductivity of deproteinized bovine bone mineral with granules of different sizes applied in maxillary sinus floor augmentation.

The null hypothesis was that of no difference in osteoconductivity or bone formation at the sites augmented with either small or large granules of deproteinized bovine bone mineral (DBBM).

## 2. Materials and Methods

The experimental protocol was submitted and approved by the Ethical Committee of the Faculty of Dentistry in Ribeirão Preto of the University of São Paulo on 14 June 2017 (USP, SP-Brazil; 2017.1.278.58.9). The study is reported according to the ARRIVE guidelines. The guidelines for animal care adopted in Brazil were strictly followed.

### 2.1. Animal Sample

Eighteen New Zealand white rabbits, approximately 3.5–4.0 kg and 4–5 months of age, were used. Three groups of six animals each were randomly assigned to a different period of healing, i.e., 2, 4, and 8 weeks, respectively.

To adhere to the Three R requirements for animal research, a rabbit model was selected owing to the simplicity of the surgical treatment. Moreover, the use of a split-mouth design reduced the variability among animals, a fact that decreased the number of animals needed. Nevertheless, for sample calculation, data from an experiment in minipigs [17] were used. In that experiment, a difference of 9.1% in BGC was found after 6 weeks of healing in favor of small compared with large granule sites. With a standard deviation of 6%, six rabbits were calculated to be sufficient to reject the null hypothesis with a power of 0.8 and an α = 0.05.

### 2.2. Randomization and Allocation Concealment

The randomization for the placement of xenograft granules of different dimensions was performed digitally (www.randomization.com, accessed on 1 July 2017) by one author not involved in the surgeries (DB). Blinding was not possible due to the visible differences between the two biomaterials, neither for the surgeon nor for the histological assessor. To limit the inclusion of biases, the surgeon (ERS) was informed about the side (right or left) on which to place the xenografts after the elevation of both sinuses. Moreover, no indications were reported on the histological slides regarding test and control sites.

### 2.3. Surgical Procedures

A maxillofacial surgeon specialist (ERS) performed all surgeries. The anesthesia was induced using acepromazine (1.0 mg/kg; Acepran^®^, Vetnil, Louveira, São Paulo, Brazil), administrated subcutaneously, and a mix of xylazine (3.0 mg/kg; Dopaser^®^, Hertape Calier, Juatuba, Minas Gerais, Brazil) and 50 mg/kg ketamine hydrochloride (Ketamin Agener, União Química Farmacêutica Nacional S/A, Embu-Guaçú, São Paulo, Brazil) injected i.m. Local anesthesia was added in the experimental regions.

**Figure 1:**
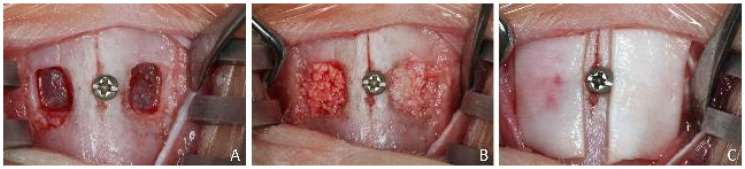
View of the clinical procedures in the experimental region. (**A**) A 3.5–4 mm antrostomy was prepared on both sides, laterally to the nasal-incisal suture, and anteriorly to the nasal-frontal suture. (**B**) Deproteinized bovine bone mineral granules either 0.250–1.0 mm or 1.0–2.0 mm were randomly allocated and grafted within the elevated space in similar quantity. (**C**) The antrostomies were subsequently covered with collagen barriers.

After having shaved and disinfected the experimental area, an incision ~2.5 cm long was carried out along the midline of the nasal dorsum. Skin, muscles, and periosteum were elevated, and the nasal bone was exposed bilaterally at the nasal-incisal suture. A squared antrostomy, of about 4 mm in dimensions, was prepared with diamond drills on both sides, laterally to the nasal-incisal suture, and anteriorly to the nasal-frontal suture (Figure 1A). The sinus mucosa was detached from the bony walls and elevated at both sides. A small screw was placed in the nasal-incisal suture as a landmark for the histological processing to identify the central position of the antrostomies. Deproteinized bovine bone mineral (DBBM) granules (Bio-Oss^®^, Geistlich Biomaterials, Wolhusen, LU, Switzerland), either 0.250–1.0 mm or 1.0–2.0 mm, were randomly allocated and grafted within the elevated space in similar volumes (Figure 1B). The antrostomies were subsequently covered with collagen barriers (Bio-Gide^®^ Geistlich Biomaterials, Wolhusen, LU, Switzerland) (Figure 1C).

Resorbable sutures were used for the periosteum (Polyglactin 910 5-0, Vicryl^®^, Ethicon, Johnson & Johnson, São José dos Campos, Brazil) while nylon sutures were used to close the skin flaps (Ethilon 4-0^®^, Ethicon, Johnson & Johnson, São José dos Campos, Brazil).

### 2.4. Maintenance Care and Euthanasia

Each animal was kept in an individual cage and within an acclimatized room. The wounds and the biological functions were carefully monitored by veterinarians during the full period of the experiment. The animals had access to food and water ad libitum.

The same procedures used to induce anesthesia during surgery were also applied for euthanasia. An overdose of sodium thiopental (1.0 g, 2 mL; Thiopentax^®^, Cristália Produtos Químicos Farmacêuticos, Itapira, São Paulo, Brazil) was added to euthanize the animals. Biopsies were retrieved in blocks and were fixed in 10% buffered formalin.

### 2.5. MicroCT Evaluations

A microCT analysis was performed using a microCT 1172 equipment (Bruker, Kontich, Belgium). The parameters were as follows: 9.92 µm isotropic pixel, 60 KV/165 µA, filter Al 0.5 mm, exposure time 596 ms, rotation step 0.4 degrees, frame average 4, and random movement 10. The software DataViewer^®^ (Bruker, Kontich, Belgium) was used to reposition the cross-sectional images, and measurements were performed with the software CTan (Bruker, Kontich, Belgium). All evaluations were performed by a calibrated author (KAAA).

### 2.6. Histological Preparation

The experimental region was reduced, and the biopsies were dehydrated in increasing concentrations of ethanol. Subsequently, the biopsies were infiltrated in resin (LR White™ hard grid, London Resin Co Ltd., Berkshire, UK). After polymerization, two ground sections were prepared using the small screw as a reference and following a transverse plane.

The ground sections were first prepared at a width of about 100–150 μm using a precision slicing equipment (Exakt^®^, Apparatebau, Norderstedt, Germany), and then they were ground to about 50–60 μm using a cutting–grinding machine (Exakt^®^, Apparatebau, Norderstedt, Germany). The sections were stained with either toluidine blue or Stevenel’s blue and alizarin red.

### 2.7. Calibration for Histomorphometric Evaluations

All histological measurements were made by a trained assessor (KAAA) after a calibration with another professional (DB) performed until the inter-rater agreement in the recognition of the tissues reached K > 0.90.

### 2.8. Histomorphometric Evaluations

The histological measurements were carried out using the software NIS-Elements D (v 4.0, Laboratory Imaging, Nikon Corporation, Tokyo, Japan) on an Eclipse Ci microscope (Nikon Corporation, Tokyo, Japan) equipped with a video camera (Digital Sight DS-2Mv, Nikon Corporation, Tokyo, Japan).

The area of the augmented space was evaluated in all three periods of healing while the residual defects on the antrostomy were measured at 8 weeks of healing.

**Figure 2:**
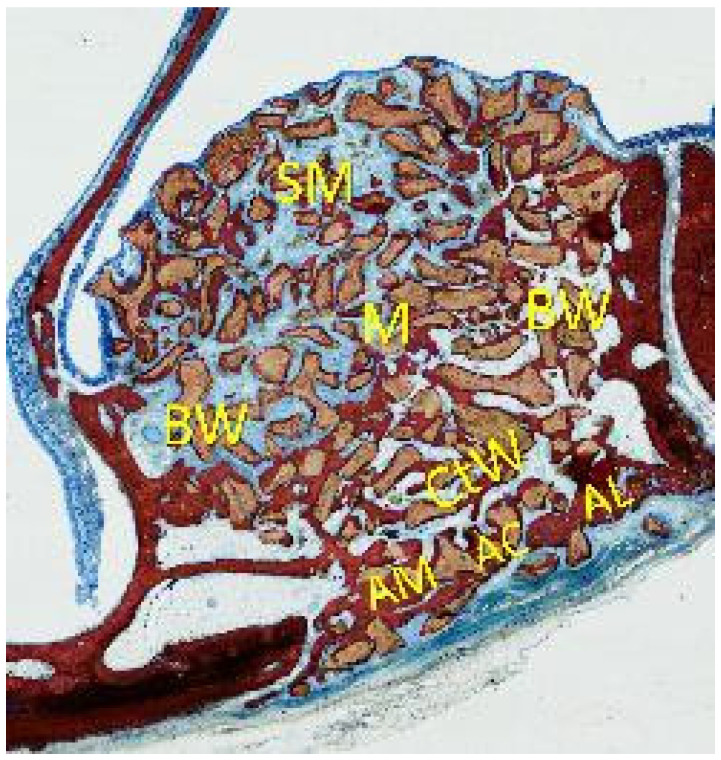
The various regions analyzed in the augmented sinus (BW = bone walls; M = middle; SM = sub-mucosa; CtW = close-to-window) and the antrostomy region (AL = lateral; AM = medial margin; AC = center of the antrostomy).

The following regions within the augmented space were analyzed (Figure 2): (i) close to the medial and lateral bony walls (bone wall regions), (ii) in the center of the elevated space (middle region), (iii) subjacent to the sinus mucosa (sub-mucosal region), and (iv) in close vicinity to the antrostomy, still within the sinus (close-to-window region). The antrostomy (antrostomy region) was evaluated in three different zones: close to the lateral and medial margins and in the center of the antrostomy.

As linear measurements, the following tissues in contact with the xenograft surface were evaluated for all granules within the evaluated regions at a magnification of ×100: mineralized bone, marrow spaces, dense and lose matrix tissues, osteoclasts, and vessels. Bone-to-implant contact percentage (BGC%) represented the proportion of bone in contact with the xenograft granule surface in relation to the total surface of the xenograft evaluated.

To perform morphometric measurements, a point-counting procedure was used [23]. Lattices with squares of 75 µm in dimensions were superposed onto the image of the histological slide at ×100 magnification. The proportions of the following tissues were included in the analyses: mineralized bone, marrow spaces, dense and lose matrix tissues, connective tissue, xenograft, inflammatory cells, osteoclasts, vessels, and membrane residues.

Moreover, the area of the augmented sinus and the residual defects in the outer part of the antrostomy were measured.

As an explorative aim, the intersection point [24] was evaluated between dense tissue and newly formed bone in contact with the graft surface.

### 2.9. Data Analysis

A report on the comparison of the merged data of both granule sizes regarding only the proportions of new bone and DBBM granule residues in histological and microCT analyses has been already published [25]. In the present study, the primary outcome variable was the osteoconductivity as expressed by BGC% as total data and for the single regions evaluated. The content in percentages of new bone, of DBBM and the other tissues, is reported separately for small and large granules. For the microCT analysis, only the volumetric changes over time are reported.

The total mineralized bone was used as a secondary variable. Mean values and standard deviations are reported for each outcome. Mean values were obtained for the two histological slides. All calculations were carried out using the software Excel 2013 (Microsoft Corporation, Redmond, WA, USA). Statistical analyses were performed for both primary and secondary variables using the IBM SPSS Statistics software (IBM Inc., Chicago, IL, USA). The Wilcoxon test was used to evaluate differences between large and small particle sizes. The level of significance was set at 5%.

## 3. Results

No perforations of the mucosa were noticed during the surgical procedures. All biopsies were collected and histologically processed, and an *n* = 6 was achieved for each period of healing.

**Figure 3:**
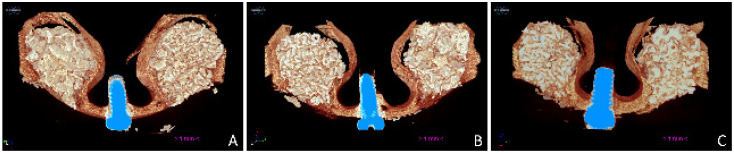
MicroCT 3D images representing the augmented sinus at the 0.125–1 mm and 1–2 mm size granules after 2 (**A**), 4 (**B**), and 8 (**C**) weeks of healing.

The volumes evaluated in the microCT (Figure 3A–C) were about 132, 114, and 115 mm^3^ at the sites with large granules, and 123, 104, and 118 mm^3^ at the sites with small granules after 2, 4, and 8 weeks, respectively. No statistically significant differences were found between large and small granule sites for any of the three healing periods analyzed.

**Figure 4:**
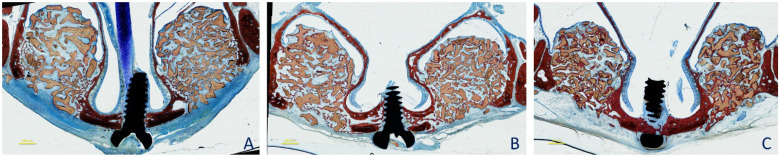
Ground sections representing the augmented sinus at the 0.125–1 mm and 1–2 mm size granules after 2 (**A**), 4 (**B**), and 8 (**C**) weeks of healing. Images originally taken with objective ×10. Stevenel’s blue and alizarin red stain.

The histological analyses (Figure 4A–C) showed that the augmented area slightly decreased over time in both augmentation sites, by 17.5 ± 3.8, 16.5 ± 2.0, and 14.6 ± 1.0 mm^2^ at the small particle sites, and by 17.1 ± 2.3, 16.6 ± 2.5, and 15.3 ± 3.1 mm^2^ at the large particle sites, after 2, 4, and 8 weeks, respectively. No statistically significant differences were found between sites or between healing periods. Small residual defects in the outer part of the antrostomy were present after 8 weeks of healing in both the small (0.3 ± 0.2 mm^2^) and the large (0.4 ± 0.3 mm^2^) particle sites. No statistically significant differences were found between sites either.

**Figure 5:**
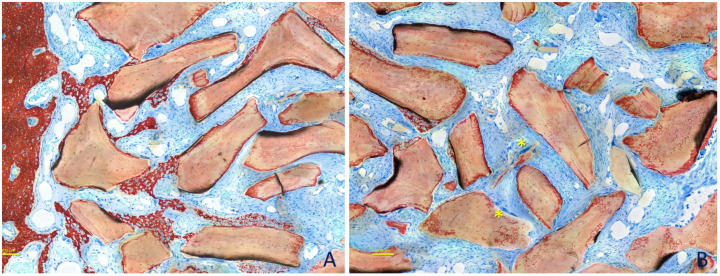
Photomicrographs of ground sections representing the healing after 2 weeks at small granules sites. (**A**) Newly formed bone was found close to the bone walls and lining on the surface of the DBBM. (**B**) A dense tissue rich in fibroblast-like cell was seen surrounding the DBBM particles. A loose tissue rich in vessels was interposed among particles. Some multicellular cells were visible (e.g., *). Original magnification ×200. Stevenel’s blue and alizarin red stain.

After 2 weeks of healing (Figure 5A,B), in the histological analyses of the content of the augmented sinuses (Table 1), bone was found at percentages of 7.0 ± 4.5% in the small granule sites and 6.3 ± 3.4% in the large granule sites (*p* = 0.686). Xenograft was occupying about 50% of the area in both sites (*p* = 0.753). Dense matrix tissue was found at about 20% in both sites (*p* = 0.917) surrounding the xenograft granules, while loose matrix tissue was interposed among the granules. New bone was found growing within the granules and in close contact with the xenograft surfaces (Table 2). The percentages of tissues in contact with the xenograft were mainly represented by dense tissue (~70%), while the newly formed bone was covering 11–12% (*p* = 0.753) of the surfaces. Bone was found at higher percentages in the bone wall regions (Table 3). Osteoclasts were found at percentages of ~5% (*p* = 0.462).

**Figure 6:**
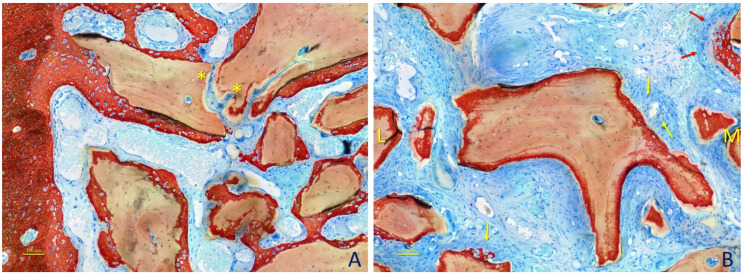
Photomicrographs of ground sections representing the healing after 4 weeks at large granules sites. (**A**) Bone walls region. Higher content of new bone was found compared with the previous period analyzed. Some multicellular cells were still visible (e.g., *). (**B**) New bone reached over time the most central regions, growing from the lateral (L) and mesial (M) bone walls of the sinus. The granule in the center presents a higher peripheral chromaticity compared with that seen in the previous period of healing. Moreover, some of the dense tissue surrounding the granule appeared to have a higher content of active cells, especially in zones where the new bone was formed between the graft surface and the cellular cluster (yellow arrows). Note a front of new bone formation and osteoid tissue (red arrows). Original magnification ×200. Stevenel’s blue and alizarin red stain.

After 4 weeks of healing (Table 1; Figure 6A,B), newly formed bone was occupying 17–18% (*p* = 0.173) of the areas analyzed, and xenograft was still occupying ~44–49% of this area (*p* = 0.116). The dense tissue proportions decreased to ~13% (*p* = 0.917). The tissues in contact with the xenograft surfaces (Table 2) were now mainly represented by newly formed bone (~49%; *p* = 0.753) while the dense tissue proportions were reduced to ~35–36% (*p* = 0.674). Osteoclast proportions also decreased to ~2% (*p* = 0.395).

**Figure 7:**
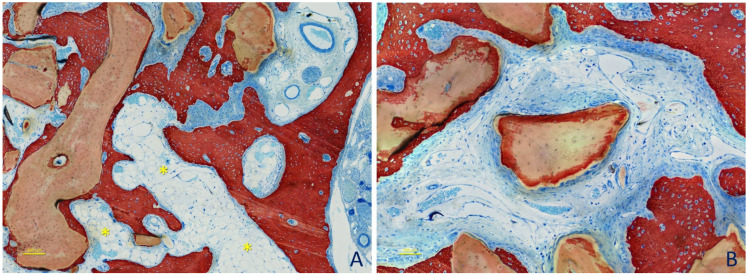
Photomicrographs of ground sections representing the healing after 8 weeks at small granule sites. (**A**) Marrow spaces were seen at this stage of healing (yellow asterisks). (**B**) A particle with augmented chromaticity, not yet reached by newly formed bone. Original magnification ×200. Stevenel’s blue and alizarin red stain.

After 8 weeks of healing (Table 1; Figure 7A,B), the new bone within the augmented area further increased to ~28% (*p* = 0.753) in both the small and large granule sites, while the dense tissue was reduced to 4–6% (*p* = 0.173). The percentages of xenograft were similar to those of the previous periods of healing. New bone in contact with the xenograft (Table 2) was coating 65 ± 7.3% of the surface at the small granule sites and 62 ± 8.7% at the large granule sites (*p* = 0.345). The corresponding percentages of dense tissue were 15.0 ± 8.1% and 21.0 ± 10% at the small and large granule sites, respectively. Osteoclasts were found at percentages <1%.

**Figure 8:**
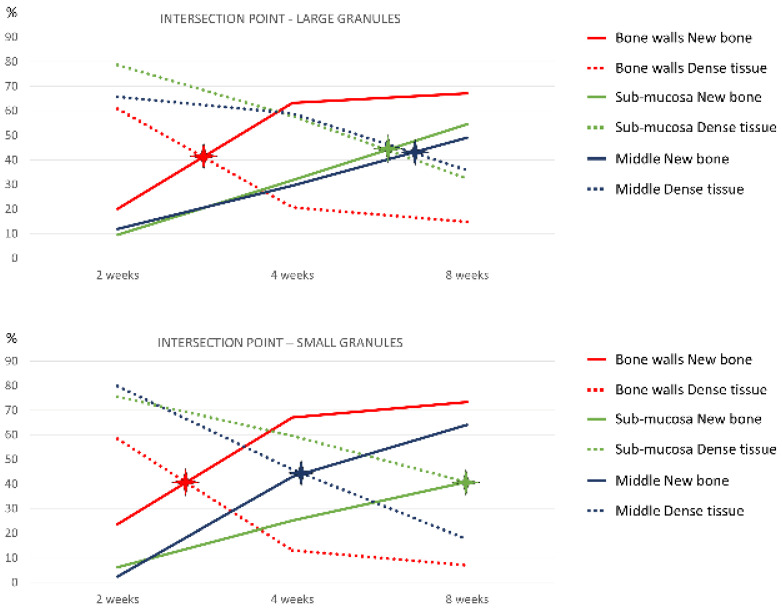
Graphs representing the percentages of BGC and dense tissue in contact with the graft surface in the various regions and periods evaluated. Stars indicate the intersection point between new bone and dense tissue.

In both granule augmentation sites, the intersection point between new bone and dense tissue (Table 4) occurred earlier in the bony wall region and later in the Schneiderian membrane region (Figure 8).

## 4. Discussion

The present experiment aimed to evaluate the osteoconductivity of deproteinized bovine bone mineral (DBBM) with granules of different sizes used for maxillary sinus floor augmentation. As a secondary aim, bone formation within the augmented sites was evaluated. No differences were found in terms of new bone formation or BGC.

The novelty of the present study was the sequential evaluation of BGC in various regions within the sinus for both small and large granules. Moreover, various tissues not analyzed in other studies that compared small and large granules were taken into consideration both within the elevated sinuses and in contact with the graft granules.

After 2 weeks of healing, the tissues in contact with the DBBM surfaces were represented mainly by dense tissue, which was surrounding up to about 70% of the surfaces of both grafts, while new bone represented 11–12%. The highest amounts of BGC were seen close to the bony walls (20–24%) in both augmentation sites. This is in agreement with an experimental study in minipigs [26], which showed a gradient of higher graft incorporation in the region close to the bone walls.

In the present study, the new bone in contact with the xenograft increased during the following periods of healing reaching fractions of 62–65% after 8 weeks, while the dense tissues were reduced correspondingly. These outcomes are in agreement with those of another study in rabbits in which small DBBM granules or a collagen sponge were used to augment the sinus floor in rabbits. In that study, healing was evaluated after 7, 14, 21, and 40 days [11]. After 40 days of healing, 68.1% of BGC was found. It was also shown that the highest bone contact to the DBBM granules in the early phases of healing was located close to the bony walls. Moreover, when the data representing the percentage of total bone (mineralized bone and marrow spaces) and that of the soft tissues (dense and loose tissues) were illustrated in a graph, the lines representing the percentages in the various periods analyzed were intercepting each other at different periods. The earliest intersection occurred in the regions close to the bony walls followed by the middle and the sub-mucosal regions. This may be interpreted as if bone formation on the DBBM surfaces started from the bony walls and then proceeded towards the other regions owing to the osteoconductive properties of the biomaterial. In the present study, similar graphs were also prepared, however, using only the data of new bone and dense tissues. At the small granule sites, analogous outcomes to the previous study [11] were observed. However, for the large granule sites, a delayed intersection point for the middle region was found compared with the small granule sites, so that the intersection point was similar to that observed in the sub-mucosa region. This, in turn, may indicate a higher osteoconductivity of small versus large DBBM particles in the central portion of the grafted region. Nevertheless, the total amount of BGC in all regions was similar. These findings are not in agreement with those of a study in minipigs [17] in which large or small DBBM particles, similar to those applied in the present experiment, were used for maxillary sinus floor augmentation. Implants were immediately installed, and the animals were euthanized after 6 or 12 weeks. The full augmented area was analyzed, and a higher proportion of BGC was observed at the small granule compared with the large granule sites in the early phases of healing.

In the present study, within the augmented area, the pattern of healing of the various tissues examined at the various periods of healing was similar in both augmentation sites. New bone increased in both sites from 6–7% to about 28% between 2 and 8 weeks of healing. During the same period of observation, the graft was reduced in percentage from about 51–53% to 44–46%. These findings support those reported by other experimental studies, which showed an increased bone formation and a decreased percentage of deproteinized bovine bone mineral (DBBM) graft over time [11,17,18].

Histological evaluation of the healing at the maxillary sinus augmented with either small or large granules of DBBM was performed both in experimental [17] and clinical studies [8,16,22].

In a minipig experiment [17], the fraction of new bone found after 6 weeks was 39.0% for the small granule sites, and 40.0% for the large granule sites. These fractions increased to 44.3% and ~45.1% for the small and large granule sites after 12 weeks, respectively. In turn, the percentages of DBBM decreased from 25.1% to 21.3% for the small granule sites, and from 24.6% to 19.8% for the large granule sites. In agreement with that study, the results of the present study allow the conclusion that the size of the granules did not influence new bone formation or DBBM degradation in augmented sites.

In a randomized clinical study [22], after 6 to 9 months of healing, the fraction of bone and DBBM was 28.0% and 34.6% at the small granule and 27.1% and 33.7% at the large granule sites, respectively. No differences were found between sites.

In another RCT study [16], 10 patients were recruited for a bilateral sinus floor elevation. After 8 months of healing, the new bone fraction was 36.1% and 23.8% and the DBBM fraction was 32.3% and 38.6%, at the small and large granule sites, respectively. Again, the differences were not statistically significant.

However, in another multicenter RCT [8], different results were obtained for the fraction of new bone. After 6–8 months, biopsies were collected from 11 patients. The small granule sites presented with 18.8% of new bone and 21.7% of DBBM. The large granule sites displayed significantly higher proportions of new bone of 26.8% and 20.0% of DBBM. Obviously, the biopsies retrieved did not represent the entire spectrum of the augmented area. Moreover, the results of the observation period of that study cannot be compared with the results of the present study.

The main limitations of the present study are the use of phylogenetically lower animals than humans, the small sample size for each period of healing analyzed, and the small dimensions of the sinuses compared with those of the xenograft granules. Moreover, the mucosal width is thinner (~0.08 mm) [12] compared with that of humans (~0.45–1 mm in histological examination [27] and ~0.9–3.1 mm in CBCT examination [28]). These limitations suggest that any inference to similar clinical situations in humans should be considered with care. Nevertheless, the rabbit model presents sinus and nasal pressure values similar to humans for absolute pressures and synchronicity with the respiratory cycle [29].

## 5. Conclusions

In conclusion, the results of the present study show that similar osteoconductive performances and similar proportions of new bone were observed in various regions of the sinus augmented with either small or large xenograft granules. Hence, both small and large granules of DBBM xenografts may be recommended for sinus floor augmentation.

## Figures and Tables

**Table 1:** Tissue components within the augmented sinuses in the three periods of evaluation. Mean values (in bold) ± standard deviations; median in percentages.

		New Bone	Marrow Spaces	DBBM	Dense Tissue	Loose Tissue	Vessels	Other Tissues
2 weeks	Small granules	**7.0** ± 4.5; 6.6	**2.4** ± 2.9; 1.8	**50.6** ± 6.4; 51.5	**20.2** ± 3.6; 20.1	**12.9** ± 5.2; 12.8	**2.9** ± 2.1; 2.6	**4.0** * ± 7.1; 1.2
	Large granules	**6.3** ± 3.4; 5.4	**3.2** ± 1.8; 2.7	**52.6** ± 7.9; 52.4	**20.3** ± 2.5; 19.7	**14.6** ± 4.9; 15.9	**2.6** ± 2.1; 2.1	**0.4** * ± 0.2; 0.3
4 weeks	Small granules	**16.7** ± 3.4; 16.9	**11.3** ± 9.8; 8.3	**48.5** ± 4.7; 47.5	**12.8** ± 7.8; 12.2	**6.4** ± 3.6; 6.7	**3.8** ± 1.4; 4.0	**0.3** ± 0.4; 0.2
	Large granules	**18.4** ± 6.0; 18.4	**11.1** ± 7.4; 9.5	**43.5** ± 3.2; 42.1	**12.9** ± 5.7; 11.6	**8.3** ± 2.3; 8.3	**5.4** ± 2.2; 4.4	**0.3** ± 0.3; 0.2
8 weeks	Small granules	**27.6** ± 4.6; 27.2	**16.4** ± 5.0; 16.3	**46.3** ± 3.4; 46.1	**6.3** ± 4.4; 5.7	**2.5** ± 2.0; 2.6	**0.7** ± 0.5; 0.5	**0.1** ± 0.3; 0.0
	Large granules	**27.6** ± 4.8; 27.0	**19.2** ± 3.8; 18.6	**44.3** ± 5.0; 43.8	**4.2** ± 3.4; 5.1	**3.1** ± 4.0; 1.2	**1.3** ± 0.7; 1.3	**0.3** ± 0.5; 0.0

*p < 0.05 between test and control sites.

**Table 2:** Tissues in contact with the DBBM surface in the three periods of evaluation. Mean values (in bold) ± standard deviations; median in percentages.

		New Bone	Marrow Spaces	Dense Tissue	Loose Tissue	Vessels	Osteoclasts
2 weeks	Small granules	**10.9** ± 6.3; 12.5	**1.0** ± 1.1; 0.9	**70.9** ± 9.1; 73.3	**12.6** ± 5.5; 14.1	**0.1** ± 0.2; 0.0	**4.6** ± 3.7; 3.5
	Large granules	**11.9** ± 5.9; 11.5	**1.5** ± 0.9; 1.7	**69.6** ± 7.4; 71.4	**11.5** ± 5.1; 11.4	**0.3** ± 0.5; 0.0	**5.1** ± 3.4; 3.9
4 weeks	Small granules	**48.6** ± 13.1; 46.3	**11.7** ± 10.4; 8.3	**35.0** ± 16.2; 34.4	**2.6** ± 2.2; 2.1	**0.0** ± 0.0; 0.0	**2.2** ± 2.1; 1.6
	Large granules	**49.1** ± 18.2; 48.3	**10.8** ± 9.1; 8.5	**35.5** ± 19.7; 32.7	**2.2** ± 2.2; 1.6	**0.0** ± 0.0; 0.0	**2.4** ± 2.3; 1.9
8 weeks	Small granules	**65.0** ± 7.3; 65.9	**16.9** ± 3.7; 17.7	**15.0** ± 8.1; 13.8	**2.2** ± 2.1; 1.9	**0.0** ± 0.0; 0.0	**0.9** ± 0.6; 0.9
	Large granules	**62.0** ± 8.7; 61.8	**15.6** ± 5.8; 15.0	**21.0** ± 10.0; 18.3	**0.5** ± 0.6; 0.3	**0.0** ± 0.0; 0.0	**0.9** ± 0.6; 0.8

*p* < 0.05 between test and control sites.

**Table 3:** BGC in the various regions evaluated within the augmented sinus in the three periods of evaluation. Mean values (in bold) ± standard deviations; median in percentages.

	Small Granules			Large Granules		
	2 Weeks	4 Weeks	8 Weeks	2 Weeks	4 Weeks	8 Weeks
Bone walls	**23.6** ± 14.2; 29.6	**67.0** ± 8.2; 69.8	**73.4** ± 6.2; 73.2	**20.1** ± 7.7; 17.4	**63.2** ± 19.2; 67.7	**67.1** ± 8.1; 67.2
Schneiderian	**6.2** ± 13.8; 0.0	**25.1** ± 22.1; 25.4	**40.8** ± 18.1; 45.0	**9.5** ± 12.7; 4.3	**31.7** ± 22.3; 32.9	**54.5** ± 24.9; 57.6
Middle zone	**2.4** ± 5.4; 0.0	**42.9** ± 23.1; 39.6	**64.0** ± 20.6; 70.0	**11.9** ± 15.6; 4.8	**29.6** ± 23.9; 24.1	**49.1** ± 22.0; 53.1
Close window	**3.1** ± 4.8; 0.0	**47.2** ± 15.3; 52.8	**74.8** ± 4.6; 76.9	**0.0** ± 0.0; 0.0	**52.0** ± 24.4; 60.4	**71.9** ± 8.7; 74.5
Full area	**10.9** ± 6.3; 12.5	**48.6** ± 13.1; 46.3	**65.0** ± 3.7; 65.9	**11.9** ± 5.9; 11.5	**49.1** ± 18.2; 48.3	**62.0** ± 8.7; 61.8

*p* < 0.05 between test and control sites.

**Table 4:** Intersection points between newly formed bone and dense tissue. Days (in bold) and percentage of BGC are reported.

	Bone Walls	Schneiderian	Middle Zone	Close Window	Full Area
Small granules	**19.5**; 40.6%	**55.9**; 40.8%	**28.6**; 44.5%	**26.1**; 41.1%	**25.4**; 41.6%
Large granules	**20.8**; 41.2%	**43.2**; 44.0%	**47.3**; 43.0%	**24.6**; 39.5%	**25.3**; 42.0%

## Data Availability

The data are available on reasonable request.
